# Supplementary material for: Hierarchical Self-Assembly and Conformation of Tb Double-Decker Molecular Magnets: Experiment and Molecular Dynamics
Source: Nanomaterials (Basel). 2023 Aug 1;13(15):2232. doi: 10.3390/nano13152232 (PMC10421050; doi:10.3390/nano13152232)
Supplement: Supplementary file 1 [file nanomaterials-13-02232-s001.zip › nanomaterials-2490599-supplementary.pdf]

nanomaterials

Supplementary Materials

Hierarchical Self-Assembly and Conformation of Tb Double-Decker Molecular Magnets: Experiment and Molecular Dynamics

Patrick Lawes [1,2], Mauro Boero [1], Rabei Barhoumi [1,2], Svetlana Klyatskaya [2], Mario Ruben [2,3] and Jean-Pierre Bucher [1,*]

[1] Institut de Physique et de Chimie de Matériaux (IPCMS), Université de Strasbourg, UMR 7504, F-67034 Strasbourg, France; patrick.lawes@ipcms.unistra.fr (P.L.); mauro.boero@ipcms.unistra.fr (M.B.); rabiybarhoumi@hotmail.fr (R.B.)

[2] Institute of Nanotechnology and Institute of Quantum Materials and Technology (IQMT), Karlsruhe Institute of Technology, 76131 Karlsruhe, Germany; svetlana.klyatskaya@kit.edu (S.K.); mario.ruben@kit.edu (M.R.)

[3] Centre Européen de Science Quantique (CESQ), Institut de Science et d'Ingénierie Supramoléculaires (ISIS), Université de Strasbourg, F-67083 Strasbourg, France

* Correspondence: jean-pierre.bucher@ipcms.unistra.fr

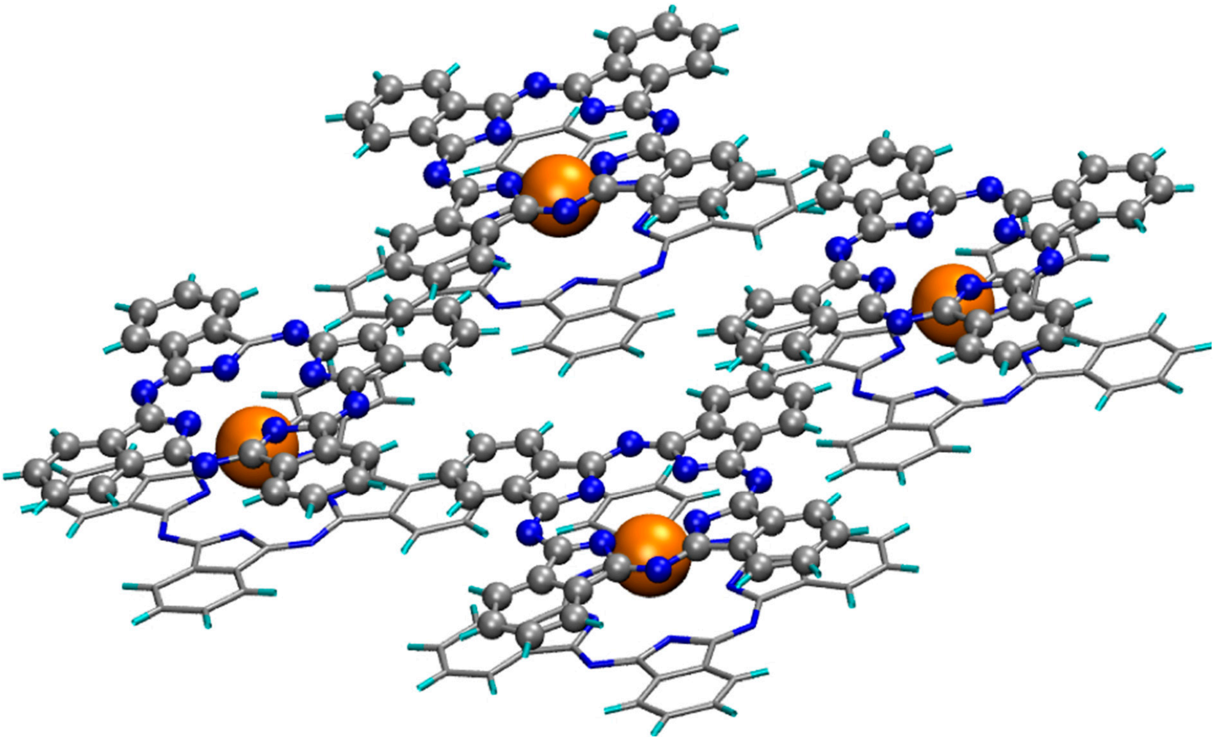

Figure S1. Perspective view of the fully relaxed $TbPc_2$ molecules. Upper Pc (balls) and lower Pc (sticks). Color code: C (grey), N (blue), Tb (orange).

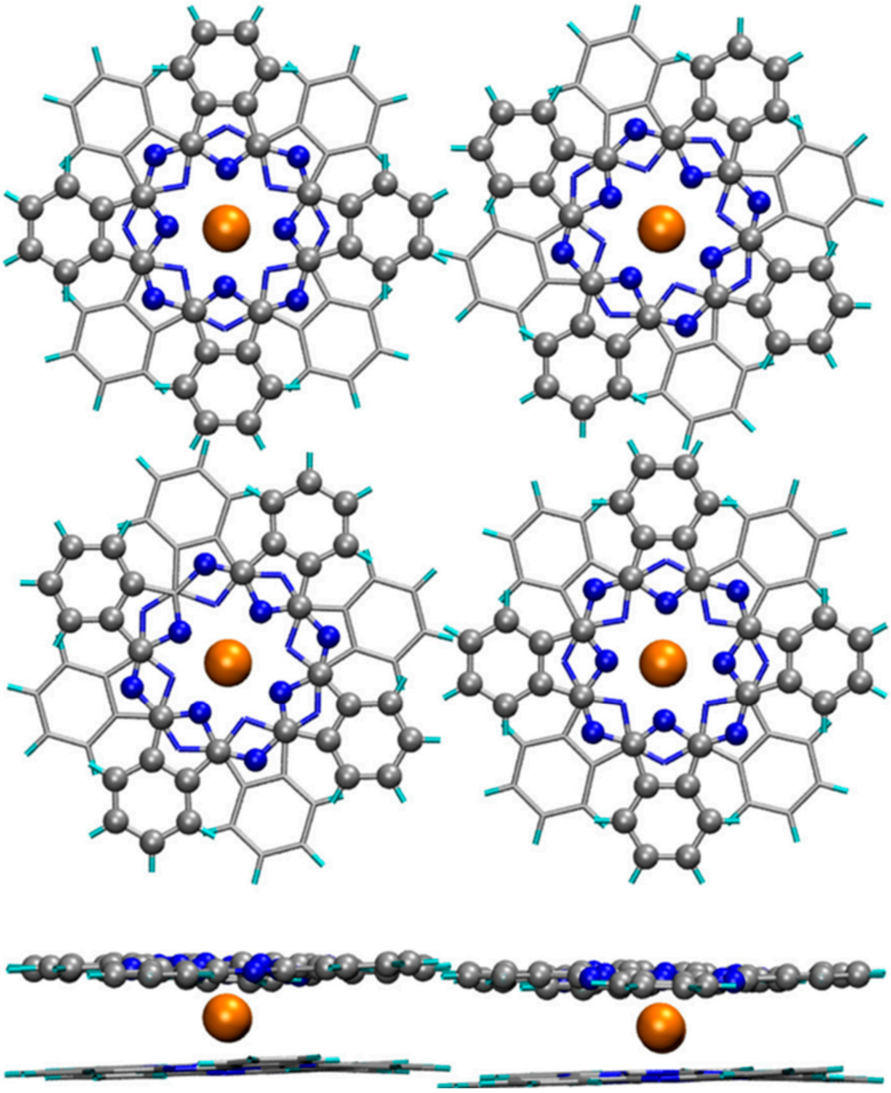

Figure S2. Assembly of four $TbPc_2$ molecules with periodic boundary conditions. The only experimental input is the dimension of the square lattice (1.42 nm). After full relaxation, it is found that ϑ = 45° for all molecules and remains unchanged, independent of initial configurations. Top view and side view. Upper Pc (balls) and lower Pc (sticks). Color code: C (grey), N (blue), Tb (orange).

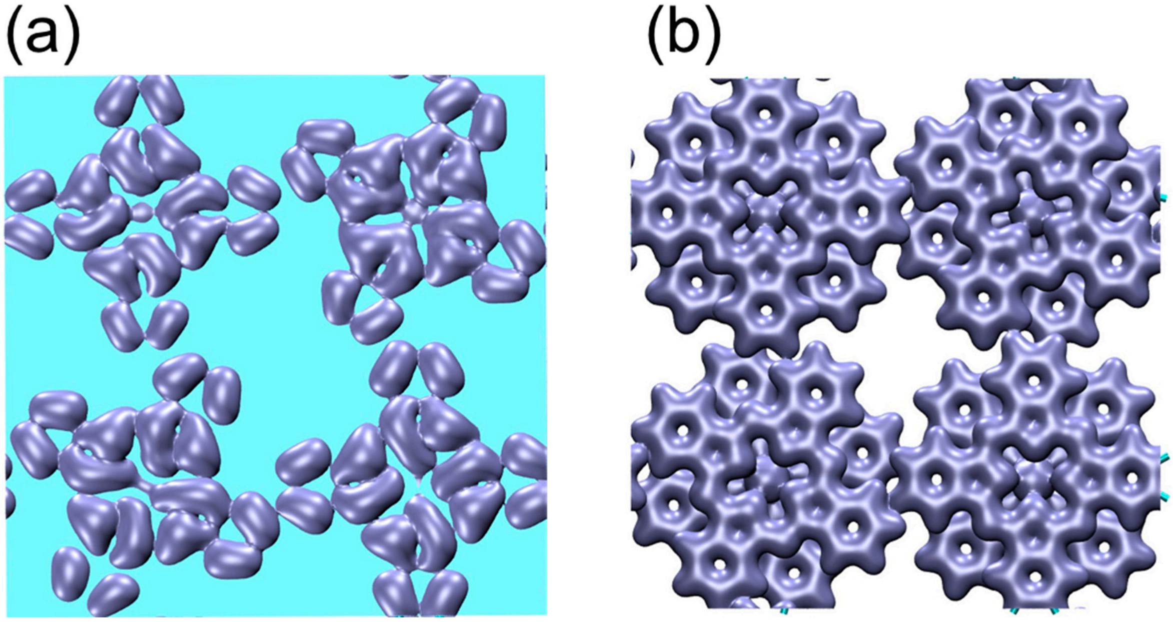

Figure S3. Assembly of four $TbPc_2$ molecules with periodic boundary conditions after full relaxation. (a) Electron density 1 x 10^{-4} e/Å^3 ; range [- 0.6 eV, 0], top view, only the upper Pc is shown for clarity. (b) Electron density 5 x 10^{-2} e/Å^3 ; range [- 3.4 eV, 0], top view, above the upper Pc. Intervals are given with respect to E_F = 0.

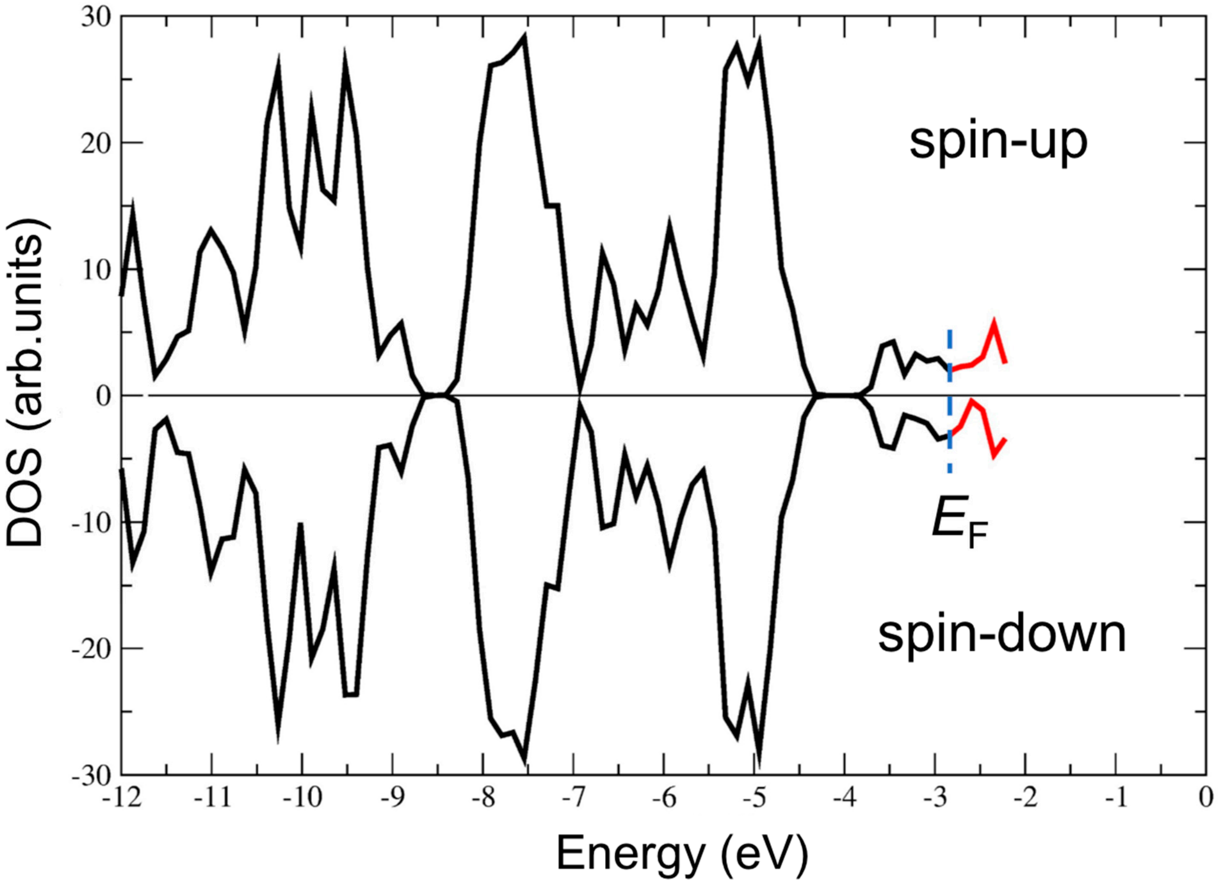

Figure S4. Density of states (DOS) for the tetramer. Occupied states (black), unoccupied states (red).
